# Rapid Scanning Electron Microscopy Detection and Sequencing of Severe Acute Respiratory Syndrome Coronavirus 2 and Other Respiratory Viruses

**DOI:** 10.3389/fmicb.2020.596180

**Published:** 2020-11-19

**Authors:** Gabriel Haddad, Sara Bellali, Anthony Fontanini, Rania Francis, Bernard La Scola, Anthony Levasseur, Jacques Bou Khalil, Didier Raoult

**Affiliations:** ^1^Aix-Marseille Université, Institut de Recherche pour le Développement(IRD), UMR Microbes Evolution Phylogeny and Infections (MEPHI), Marseille, France; ^2^Institut Hospitalo-Universitaire Méditerranée-Infection, Marseille, France

**Keywords:** SARS-CoV-2, COVID-19, virus detection, sequencing, scanning electron microscopy

## Abstract

There is an urgent need for accurate and rapid testing methods to quickly identify infected patients as well as asymptomatic carriers, in order to prevent the spread of emerging viruses. Here, we developed a rapid testing strategy by scanning electron microscopy capable of detecting severe acute respiratory syndrome coronavirus 2 (SARS-CoV-2) and other respiratory viruses directly from patients. We evaluated our results by comparing them to real-time reverse transcription-polymerase chain reaction (RT-PCR) and metagenomic sequencing results. We correlated the presence of the SARS-CoV-2 to the viral load, where samples with Ct values lower than 18 were all detected by scanning electron microscopy (SEM). The sensitivity deacresed progressively with higher Ct values. In addition, we found a correlation with metagenomic sequencing, where all samples detected by SEM were sequenced and viral sequences were easily recovered. Following this study, SEM proved its efficiency as a frontline method for directly detecting previously unknown microorganisms that cannot be targeted by molecular methods and can cause potential outbreaks.

## Introduction

Over recent decades, many viruses have been at the origin of outbreaks causing severe respiratory illness and, in some cases, deaths (Drosten et al., 2003; Zaki et al., 2012; Zhu et al., 2020). The current pandemic of the severe acute respiratory syndrome coronavirus 2 (SARS-CoV-2) redirected researchers toward the discovery and analysis of emerging viruses. One of the first methods used for viral diagnosis since the 1940s was electron microscopy (EM). EM has been a reliable tool for the classification of viruses according to their ultra-structure (Hazelton and Gelderblom, 2003; Curry et al., 2006). This pioneering method was later associated to virus isolation by cell culture (La Scola et al., 2020) and serological methods (Perera et al., 2013; Müller et al., 2015; Okba et al., 2020). However, in the last decades, the emergence of molecular tools such as real-time quantitative polymerase chain reaction has nearly replaced all previous methods to allow accurate and direct diagnosis of known viral diseases (Corman et al., 2012a,b; Amrane et al., 2020). More recently, direct nucleic acid extraction associated to next generation sequencing was used for the detection and identification directly from clinical samples (Barzon et al., 2013; Li et al., 2016). This blind sequencing strategy can be costly when the choice of samples to be sequenced is not well guided. This could be due to the lack of viral infections in some cases or to a low viral load in the samples, which is a limiting factor to sequence detection. Moreover, the presence of common infections such as with influenza virus (Ly et al., 2019), or bacterial infections such as *Haemophilus influenza* (Abat et al., 2015), can also affect the efficiency and thus increase the cost of this strategy.

Recently, we managed to detect SARS-CoV-2 viral particles directly in the nasopharyngeal swab of the first patient from whom we received a sample with a high viral load using a new generation of scanning electron microscopes (SEMs), and therefore, we succeeded to sequence (SEQ) its viral genome directly from the clinical sample (Colson et al., 2020). In this study, we adopted the same SEM-SEQ strategy in order to standardize it in a prospective way. For this, we compared the ability to detect SARS-CoV-2 in the nasopharyngeal swabs diagnosed by real-time reverse transcription-polymerase chain reaction (RT-PCR) and checked if the samples detected as positive by SEM could be directly sequenced. Our aim was first to find a direct link or a screening strategy for viral detection by SEM followed by metagenomic SEQ from the sample, which would save a lot of time and cost on the sequences. We then applied this process to other selected known viruses, suggesting that we could identify future emerging viruses by this strategy without going through culture.

## Materials and Methods

### Sample Collection

A total of 118 nasopharyngeal swab samples, collected from patients with suspected SARS-CoV-2 and tested using RT-PCR (Amrane et al., 2020), were assessed by scanning electron microscopy (SEM). These samples were also co-cultured on Vero E6 cells (ATCC CRL-1586) to isolate the virus (La Scola et al., 2020). We also collected 28 nasopharyngeal and sputum samples positive for other respiratory tract viruses from the Orthomyxoviridae family (*Influenzavirus*), the Adenoviridae family (*Adenovirus*), and the Paramyxoviridae family [*Respiratory Syncytial Virus* (RSV)], as well as samples positive for other coronavirus strains: three NL63, one E229, two OC43, and five KU1.

### Sample Preparation for Scanning Electron Microscopy

**FIGURE 1:**
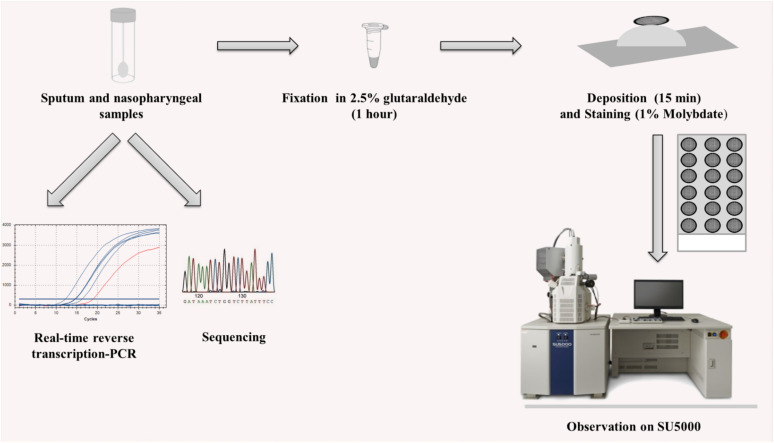
Workflow of the strategy used.

All samples were fixed for at least 1 h in 2.5% glutaraldehyde. The following steps were then carried out under a biosafety level 2 hood. First, the carbon grids underwent glow discharge for 2 min. A carbon grid was deposited on top of 10–30 μl of the fixed sample for 15 min. The grids were then instantly stained with a 1% molybdate solution. Eighteen contrasted carbon grids were then loaded onto a multi-well glass slide using double-sided conductive adhesive tape and sputtered with a 5-μm-thick platinum layer using Ion Sputter MC1000 in order to reduce charging of the non-conductive samples. The assay workflow is illustrated in Figure 1.

### Image Acquisition

We used the Hitachi SU5000 scanning electron microscope (SEM) (Hitachi High-Tech Corporation, Toranomon Hills Business Tower, 1-17-1 Toranomon, Minato-ku, Tokyo 105-6409, Japan) for image acquisition. This SEM allows specimen observation under both low and high vacuum pressure with a short evacuation time after specimen loading. The evacuation time after loading specimens into the SEM chamber is less than 6 min. Micrographs were acquired at magnifications ranging from × 10,000 to × 70,000, with 10 and 15 kV voltages and a spot intensity of 30 using the BSE detector. All acquisition settings are displayed on the generated micrographs.

### Criteria for Viral Particles Detection

**FIGURE 2:**
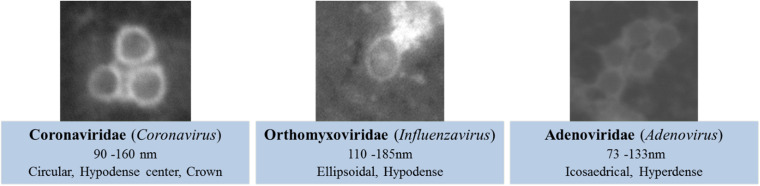
Morphological criteria for viral detection on scanning electron microscopy (SEM)-acquired micrographs.

We used viral co-culture supernatants of each virus to correlate the observed morphological structures to the different viral particles. These samples underwent the same experimental procedures as previously described for the clinical samples. We targeted the morphology, size, and electron density of the imaged particles and assigned the different criteria (also described in the literature; Goldsmith and Miller, 2009; Zhu et al., 2020) to the acquired micrographs (Figure 2). Acquired micrographs were blindly analyzed by two different operators.

### Extraction, SEQ, Data Processing, and Annotation

Genomic DNA was extracted using the EZ1 BioRobot with the EZ1 DNA Tissue Kit (Qiagen, Hilden, Germany). For RNA viruses, in the reverse transcription (RT) step, cDNA was reverse transcribed from total viral RNA samples using TaqMan Reverse Transcription (Life Technologies Applied Biosystems) following the manufacturer recommendations. PCR program was set as follows: one step at 25°C for 10 min, one step at 48°C for 30 min, one step at 95°C for 5 min and then at 4°C (Supplementary Table S1). DNA Polymerase I Large Klenow Fragment (BioLabs) was used for generating double-stranded cDNA. A 20 μl volume of each sample was added to a mix (10 μl) of the following: NEBuffer 2 10X, DNA Polymerase Large Klenow Fragment, and dNTP working solution (10 mM) for 1 h at 37°C. cDNA were purified by using beads Agencourt AMPure (Beckman Coulter) and then sequenced with the paired-end strategy on a MiSeq sequencer (Illumina Inc., San Diego, CA, United States) with the Nextera XT DNA Sample Prep Kit (Illumina). To prepare the paired-end library, the “tagmentation” step fragmented and tagged the DNA. Then, limited-cycle PCR amplification (12 cycles) completed the tag adapters and introduced dual-index barcodes. After purification on AMPure XP beads (Beckman Coulter Inc., Fullerton, CA, United States), the libraries were then normalized on specific beads according to the Nextera XT protocol (Illumina). Normalized libraries were pooled into a single library for sequencing (SEQ) on the MiSeq. The pooled single-strand library was loaded onto the reagent cartridge and then onto the instrument along with the flow cell. Automated cluster generation and paired-end SEQ with dual index reads were performed in a single 39 h run in 2 × 250 bp. After SEQ, reads were mapped on the human reference (GRCh38; GCA_000001405.28) using CLC Genomics Workbench v.7. All human reads were discarded, and the remaining reads were BLASTed against RefSeq viral sequences from NCBI database.

### Statistical Analysis

In order to determine the performance of SEM in detecting SARS-CoV-2 directly from samples based on RT-PCR results as reference, we calculated the correlation between the positivity of the samples by SEM to the Ct values obtained by RT-PCR. We also calculated the area under the receiver operating characteristic (AUROC) curve. Statistical analyses were performed using XLSTAT 2020.1.2 (Addinsoft, Paris, France). *P-*value < 0.05 was considered statistically significant.

## Results

### Morphological Characterization and Classification Criteria

**FIGURE 3:**
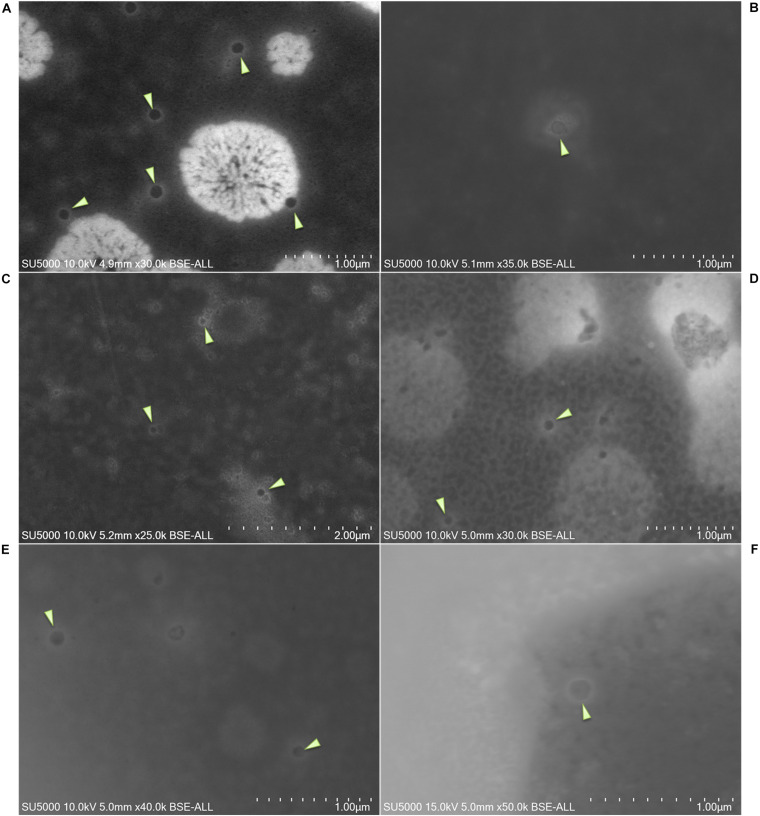
**(A–F)** Micrographs of different nasopharyngeal samples positive for severe acute respiratory syndrome coronavirus 2 (SARS-CoV-2) assessed by scanning electron microscopy. Arrows show SARS-CoV-2 particles. Scale bars are shown on the micrographs.

**FIGURE 4:**
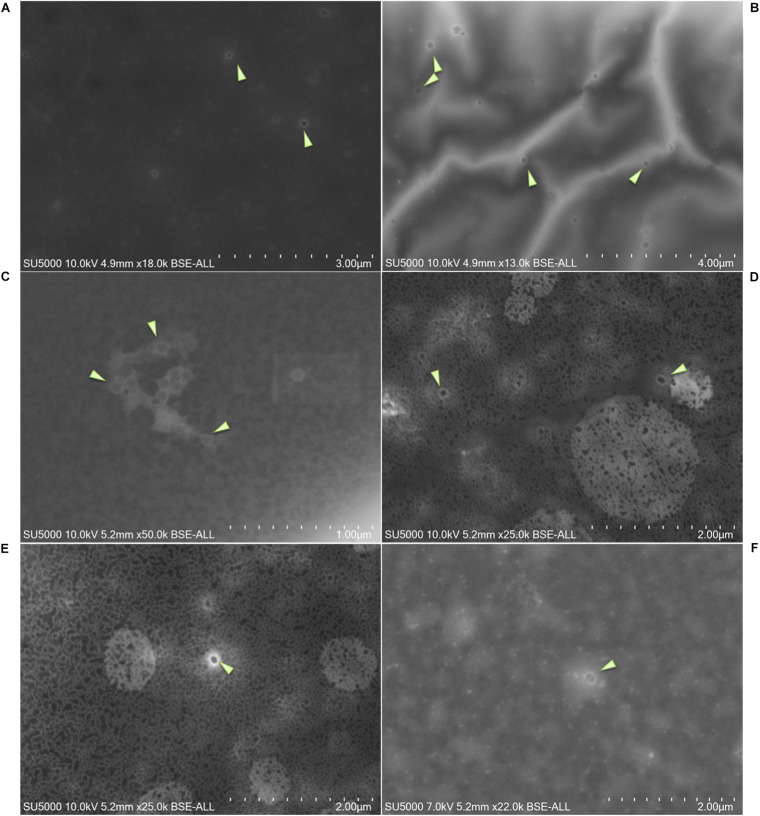
Micrographs of negative samples for SARS-CoV-2 and positive for other viruses. **(A)** NL63 *Coronavirus*; **(B)** KU1*Coronavirus*; **(C)** *Adenovirus* (size = 73–133 nm); **(D–F)** *Influenzavirus* (ellipsoidal shape, size = 110–185 nm). Arrows show viral particles. Scale bars are shown on the micrographs.

SARS-CoV-2 viral particles were hypo-electron-dense circular structures surrounded by a hyper-dense crown-like shape (Figures 2, 3). Their size ranged between 75 and 155 nm. Concerning the NL63, E229, OC43, and KU1 viral strains, we were able to identify the viral particles by SEM but were not able to differentiate them from SARS-CoV-2 particles neither by morphology nor by size (Figures 4A,B). On the other hand, the Paramyxoviridae family were differentiated from Coronaviridae according to their size (RSV = 219–224 nm), whereas the Orthomyxoviridae and Adenoviridae families presented a different morphology (*Influenzavirus* presents ellipsoidal shapes and *Adenovirus* were icosahedral) (Figures 2, 4C–F).

### PCR and SEM

**FIGURE 5:**
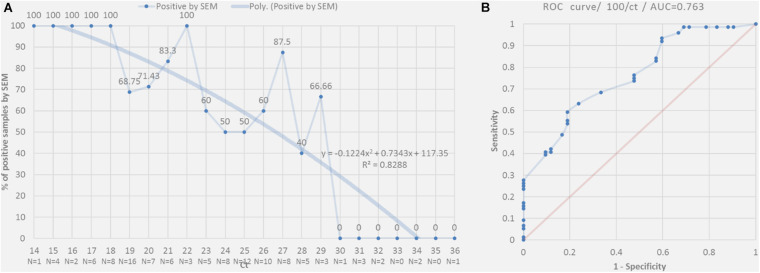
**(A)** Percentage of positive SARS-CoV-2 nasopharyngeal samples detected by SEM correlated to RT-PCR according to Ct value (plain line). The dashed curve indicates the polynomial regression curve. **(B)** Receiver operating characteristic (ROC) curves for positive samples detected by RT-PCR to predict the detection of the virus in these samples by SEM.

We were able to correlate the Ct values obtained by RT-PCR to the presence of these viral particles [*R*^2^ = 0.828] (Figure 5A). On the acquired micrographs (Figure 3) of all samples with Ct values lower than 18, we observed structures that were compatible in size and shape with those of coronavirus virions previously described (Zhu et al., 2020). We noticed a decrease in the percentage of samples detected positive by SEM with the increase of the Ct value (Figure 5A), and we were not able to detect any virus-like particles in samples with Ct ≥ 30 and in all negative samples with Ct ≥ 40. In addition, based on the results of the ROC curve analysis (Figure 5B), we found a significant (*P* < 0.0001) relationship between the Ct value and the virus detection by SEM, with 22 Ct as the threshold value for detectingSARS-CoV-2, and the area under the ROC curve was 0.76 [95% confidence interval (CI), 0.67–0.84]. By using the RT-PCR results as reference, the sensitivity and the specificity of SEM in diagnosing SARS-CoV-2 were 59% (95% CI, 48–69%) and 81% (95% CI, 66–90%), respectively. In addition, we noticed that we had less viral particles in the samples containing viruses other than SARS-CoV-2 (Coronaviridae, Orthomyxoviridae, and Adenoviridae), which correlated with their high Ct values.

### SEQ and SEM

**FIGURE 6:**
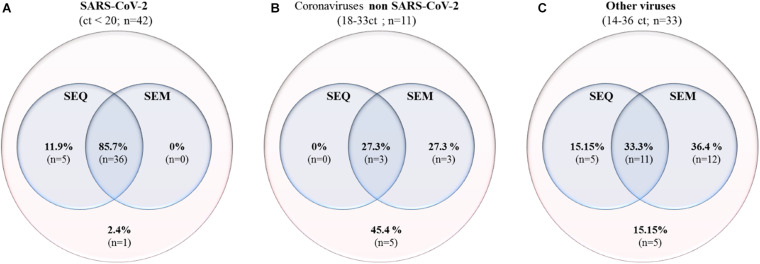
Venn diagrams comparing the detection by SEM and by SEQ of SARS-CoV-2 samples with Ct < 20 **(A)**, other Coronavirus strains (NL63, KU1, E229, and OC43) **(B)**, and other virus families [Orthomyxoviridae (Influenzavirus) and Adenoviridae (Adenovirus)] **(C)**.

In order to correlate the SEM detection and the SEQ results, a total of 86 strains were sequenced. Regarding the SARS-CoV-2 swabs, only the samples with Ct values lower than or equal to 20 were sequenced. The choice was based on a preliminary study on 1,352 sequenced samples (unpublished data). A sequence-based identification for each strain was performed based on similarity searches. For the SARS-CoV-2, 85.7% were identified by both SEM and SEQ, 11.9% were only identified by SEQ, and finally, 2.4% were not detected by either method (Figure 6A). Concerning the other Coronaviridae, 27.3% were identified by both SEM and SEQ, 27.3% were identified only by SEM, and 45.4% strains were not detected by either method (Figure 6B). Concerning the other viruses, 33.3% were identified by both SEM and SEQ, 15.15% were only identified by SEQ, 36.4% were identified only by SEM, and 15.15% were not detected by either method (Figure 6C).

## Discussion

Recently, a variety of suitable assays have been made available to test the presence of SARS-CoV-2 in suspected patients, notably RT-PCR which is currently considered to be the gold standard method for the diagnosis of SARS-CoV-2 (Corman et al., 2020). However, other procedures such as SEM, cell culture, and direct viral genome SEQ using next generation sequencing were used (Yongfeng et al., 2011; Colson et al., 2020). A recent study correlated RT-PCR Ct values and successful virus isolation by co-culture (La Scola et al., 2020), where the virus viability in co-culture decreased with increasing Ct values. Similar results were obtained using our SEM-SEQ detection strategy. In the present study, we developed the association of SEM-SEQ as a new rapid strategy for the detection of SARS-CoV-2 as a paradigm for an alternative method of diagnosis of known and unknown emerging respiratory viruses. This strategy appears as the perfect complement of direct viral sequencing proposed as the best strategy for the diagnosis of emerging viral diseases, especially for unknown viruses (Goldsmith and Miller, 2009).

The use of equipment such as the SU5000 used in this work considerably facilitates the analysis and allows a very significant time saving compared to other electron microscopes that require longer sample preparation times (e.g., resin embedding, sectioning, contrast, etc.). Furthermore, a total of 54 samples can be loaded simultaneously into the microscope chamber, and thus, metal deposition and vacuum are performed only once. Another major advantage of SEM over molecular and serological techniques in the diagnosis of viruses is that it provides an open view on the sample, therefore detecting any microorganism suspected of being pathogenic. This strategy could be limited by the availability of a sufficient sample volume, making it harder to detect and identify the viral particles by SEM, which also requires trained and experienced personnel. Eventually, in the future, the constitution of an image database allowing the identification of viral families with the help of artificial intelligence will be able to further increase the capacity of discrimination between the different viruses. Metagenomic sequencing of samples is increasingly emerging as an alternative to multiplexed PCR techniques (Babady, 2018; Huang et al., 2018), since it can be used to identify both known and unknown viruses and bacteria. However, sequencing all the samples received for diagnosis is highly time consuming and costly, and therefore, an initial sorting step by SEM makes it possible to confirm the presence or absence of suspected microorganisms in sufficient amounts to be detected and identified by metagenomic sequencing. Herein, we provide a proof of concept to the work that was carried out during the COVID-19 pandemic, showing the effectiveness of this procedure for the SARS-CoV-2 and other respiratory viruses that we were able to test. These strategies open the door not only to new diagnostic possibilities but also to the discovery of microorganisms that remain unknown. Finally, the detection of virus by SEM can also be a cost- and time-efficient screening strategy for samples to be cultured on large panels of continuous or primary cell lines.

## Data Availability Statement

All SARS-CoV-2 sequencing data generated or analysed during this study are available on GISAID (accession Id numbers in Supplementary Table S2). All other respiratory viruses were deposited at ENA EMBL-EBI under the accession number PRJEB40900.

## Ethics Statement

This study was approved by the ethical committee of the University Hospital Institute M diterran e Infection (No. 2020-01). All subjects gave a written informed consent in accordance with the Declaration of Helsinki.

## Author Contributions

GH, SB, AF, AL, RF, and JBK did the experiments and analyzed the data. GH, SB, and JBK wrote the manuscript. DR and JBK conceived the project and supervised the experiments. BL, DR, and JBK reviewed the manuscript. All authors contributed to the article and approved the submitted version.

## Conflict of Interest

DR is a consultant in microbiology for the Hitachi High-Tech Corporation. The remaining authors declare that the research was conducted in the absence of any commercial or financial relationships that could be construed as a potential conflict of interest.

## References

[B1] Abat C. Chaudet H. Colson P. Rolain J.-M. Raoult D. (2015). Real-time microbiology laboratory surveillance system to detect abnormal events and emerging infections Marseille, France. *Emerg. Infect. Dis.* 21 1302–1310. 10.3201/eid2108.141419 26196165 PMC4517727

[B2] Amrane S. Tissot-Dupont H. Doudier B. Eldin C. Hocquart M. Mailhe M. (2020). Rapid viral diagnosis and ambulatory management of suspected COVID-19 cases presenting at the infectious diseases referral hospital in Marseille, France,-January 31st to March 1st, 2020: a respiratory virus snapshot. *Travel Med. Infect. Dis.* 36:101632 10.1016/j.tmaid.2020.101632 PMC7102626 32205269

[B3] Babady N. E. (2018). Importance of accuracy of multiplex PCR systems for rapid diagnosis of respiratory virus infection. *Clin. Microbiol. Infect* 24 1033–1034. 10.1016/j.cmi.2018.05.018 29870853

[B4] Barzon L. Lavezzo E. Costanzi G. Franchin E. Toppo S. Palù G. (2013). Next-generation sequencing technologies in diagnostic virology. *J. Clin. Virol.* 58 346–350. 10.1016/j.jcv.2013.03.003 23523339

[B5] Colson P. Lagier J.-C. Baudoin J.-P. Khalil J. B. La Scola B. Raoult D. (2020). Ultrarapid diagnosis, microscope imaging, genome sequencing, and culture isolation of SARS-CoV-2. *Eur. J. Clin. Microbiol. Infect. Dis.* 10.1007/s10096-020-03869-w [Epub ahead of print]. 32270412 PMC7138953

[B6] Corman V. Eckerle I. Bleicker T. Zaki A. Landt O. Eschbach-Bludau M. (2012a). Detection of a novel human coronavirus by real-time reverse-transcription polymerase chain reaction. *Eurosurveillance* 17:20285. 10.2807/ese.17.39.20285-en 23041020

[B7] Corman V. Müller M. Costabel U. Timm J. Binger T. Meyer B. (2012b). Assays for laboratory confirmation of novel human coronavirus (hCoV-EMC) infections. *Eurosurveillance* 17:20334. 10.2807/ese.17.49.20334-en 23231891

[B8] Corman V. M. Landt O. Kaiser M. Molenkamp R. Meijer A. Chu D. K. (2020). Detection of 2019 novel coronavirus (2019-nCoV) by real-time RT-PCR. *Eurosurveillance* 25:2000045. 10.2807/1560-7917.ES.2020.25.3.2000045 PMC6988269 31992387

[B9] Curry A. Appleton H. Dowsett B. (2006). Application of transmission electron microscopy to the clinical study of viral and bacterial infections: present and future. *Micron* 37 91–106. 10.1016/j.micron.2005.10.001 16361103 PMC7126980

[B10] Drosten C. Günther S. Preiser W. Van Der Werf S. Brodt H.-R. Becker S. (2003). Identification of a novel coronavirus in patients with severe acute respiratory syndrome. *New Engl. J. Med.* 348 1967–1976. 12690091 10.1056/NEJMoa030747

[B11] Goldsmith C. S. Miller S. E. (2009). Modern Uses of Electron Microscopy for Detection of Viruses. *CMR* 22 552–563. 10.1128/CMR.00027-09 19822888 PMC2772359

[B12] Hazelton P. R. Gelderblom H. R. (2003). Electron microscopy for rapid diagnosis of emerging infectious agents. *Emerg. Infect. Dis.* 9:294. 10.3201/eid0903.020327 12643823 PMC2958539

[B13] Huang H.-S. Tsai C.-L. Chang J. Hsu T.-C. Lin S. Lee C.-C. (2018). Multiplex PCR system for the rapid diagnosis of respiratory virus infection: systematic review and meta-analysis. *Clin. Microbiol. Infect* 24 1055–1063. 10.1016/j.cmi.2017.11.018 29208560 PMC7128951

[B14] La Scola B. Le Bideau M. Andreani J. Hoang V. T. Grimaldier C. Colson P. (2020). Viral RNA load as determined by cell culture as a management tool for discharge of SARS-CoV-2 patients from infectious disease wards. *Eur. J. Clin. Microbiol. Infect. Dis.* 39 1059–1061. 10.1007/s10096-020-03913-9 32342252 PMC7185831

[B15] Li D. Li Z. Zhou Z. Li Z. Qu X. Xu P. (2016). Direct next-generation sequencing of virus-human mixed samples without pretreatment is favorable to recover virus genome. *Biol. Direct.* 11:3. 10.1186/s13062-016-0105-x 26754142 PMC4710016

[B16] Ly T. D. A. Edouard S. Badiaga S. Tissot-Dupont H. Hoang V. T. Pommier de Santi V. (2019). Epidemiology of respiratory pathogen carriage in the homeless population within two shelters in Marseille, France, 2015-2017: cross sectional 1-day surveys. *Clin. Microbiol. Infect* 25 249.e1–249.e6. 10.1016/j.cmi.2018.04.032 29777925 PMC7128312

[B17] Müller M. A. Meyer B. Corman V. M. Al-Masri M. Turkestani A. Ritz D. (2015). Presence of Middle East respiratory syndrome coronavirus antibodies in Saudi Arabia: a nationwide, cross-sectional, serological study. *Lancet Infect. Dis.* 15 559–564. 25863564 10.1016/S1473-3099(15)70090-3 PMC7185864

[B18] Okba N. M. Muller M. A. Li W. Wang C. GeurtsvanKessel C. H. Corman V. M. (2020). SARS-CoV-2 specific antibody responses in COVID-19 patients. *medRxiv* [Preprint]. 10.1101/2020.03.18.20038059

[B19] Perera R. Wang P. Gomaa M. El-Shesheny R. Kandeil A. Bagato O. (2013). Seroepidemiology for MERS coronavirus using microneutralisation and pseudoparticle virus neutralisation assays reveal a high prevalence of antibody in dromedary camels in Egypt, June 2013. *Eurosurveillance* 18:20574. 10.2807/1560-7917.es2013.18.36.20574 24079378

[B20] Yongfeng H. Fan Y. Jie D. Jian Y. Ting Z. Lilian S. (2011). Direct pathogen detection from swab samples using a new high-throughput sequencing technology. *Clin. Microbiol. Infect* 17 241–244. 10.1111/j.1469-0691.2010.03246.x 20412188 PMC7129681

[B21] Zaki A. M. Van Boheemen S. Bestebroer T. M. Osterhaus A. D. Fouchier R. A. (2012). Isolation of a novel coronavirus from a man with pneumonia in Saudi Arabia. *New Engl. J. Med.* 367 1814–1820. 10.1056/nejmoa1211721 23075143

[B22] Zhu N. Zhang D. Wang W. Li X. Yang B. Song J. (2020). A novel coronavirus from patients with pneumonia in China, 2019. *New Engl. J. Med.* 382 727–733. 31978945 10.1056/NEJMoa2001017 PMC7092803

