# Expression of Concern: Rapid scanning electron microscopy detection and sequencing of severe acute respiratory syndrome Coronavirus 2 and other respiratory viruses

**DOI:** 10.3389/fmicb.2025.1656750

**Published:** 2025-07-07

**Authors:** Frontiers Editorial Office

Following publication, it was brought to our attention that the ethical approval for this study referred to the same ethics approval number 2020-01 that had been used across a series of published studies (1–3). Additionally, an issue around the potential need for approval from a Comité de Protection de Personnes was raised and remains unresolved.

The authors and their institution provided an explanation, claiming that the retrospective nature of the study, conducted on samples considered as waste, exempts them from the need for ethical approval. However, no position regarding the reuse of the same ethics approval number has been provided.

Frontiers has not received the necessary documentation to confirm the institution's responses.

Gautret, P., Lagier, J. C., Parola, P., Hoang, V. T., Meddeb, L., Sevestre, J., et al. (2020). Clinical and microbiological effect of a combination of hydroxychloroquine and azithromycin in 80 COVID-19 patients with at least a six-day follow up: A pilot observational study. *Travel Med. Infect. Dis*. 34:101663. doi: 10.1016/j.tmaid.2020.101663

Francis, R., Le Bideau, M., Jardot, P., Grimaldier, C., Raoult, D., Bou Khalil, J. Y., et al. (2021). High-speed large-scale automated isolation of SARS-CoV-2 from clinical samples using miniaturized co-culture coupled to high-content screening. *Clin. Microbiol. Infect*. 27, 128.e1–128.e7. doi: 10.1016/j.cmi.2020.09.018

La Scola, B., Le Bideau, M., Andreani, J., Hoang, V. T., Grimaldier, C., Colson, P., et al. (2020). Viral RNA load as determined by cell culture as a management tool for discharge of SARS-CoV-2 patients from infectious disease wards. *Eur. J. Clin. Microbiol. Infect. Dis*. 39, 1059–1061. doi: 10.1007/s10096-020-03913-9

